# Type II Transmembrane Serine Protease Gene Variants Associate with Breast Cancer

**DOI:** 10.1371/journal.pone.0102519

**Published:** 2014-07-16

**Authors:** Kaisa Luostari, Jaana M. Hartikainen, Maria Tengström, Jorma J. Palvimo, Vesa Kataja, Arto Mannermaa, Veli-Matti Kosma

**Affiliations:** 1 Institute of Clinical Medicine, Pathology and Forensic Medicine, University of Eastern Finland, Kuopio, Finland; 2 Biocenter Kuopio and Cancer Center of Eastern Finland, University of Eastern Finland, Kuopio, Finland; 3 Imaging Center, Clinical Pathology, Kuopio University Hospital, Kuopio, Finland; 4 Institute of Clinical Medicine, Oncology, University of Eastern Finland, Kuopio, Finland; 5 Cancer Center, Kuopio University Hospital, Kuopio, Finland; 6 Institute of Biomedicine, University of Eastern Finland, Kuopio, Finland; Meharry Medical College, United States of America

## Abstract

Type II transmembrane serine proteases (TTSPs) are related to tumor growth, invasion, and metastasis in cancer. Genetic variants in these genes may alter their function, leading to cancer onset and progression, and affect patient outcome. Here, 464 breast cancer cases and 370 controls were genotyped for 82 single-nucleotide polymorphisms covering eight genes. Association of the genotypes was estimated against breast cancer risk, breast cancer–specific survival, and survival in different treatment groups, and clinicopathological variables. SNPs in *TMPRSS3* (rs3814903 and rs11203200), *TMPRSS7* (rs1844925), and *HGF* (rs5745752) associated significantly with breast cancer risk (*P*_trend_ = 0.008–0.042). SNPs in *TMPRSS1* (rs12151195 and rs12461158), *TMPRSS2* (rs2276205), *TMPRSS3* (rs3814903), and *TMPRSS7* (rs2399403) associated with prognosis (*P* = 0.004–0.046). When estimating the combined effect of the variants, the risk of breast cancer was higher with 4–5 alleles present compared to 0–2 alleles (*P* = 0.0001; OR, 2.34; 95% CI, 1.39–3.94). Women with 6–8 survival-associating alleles had a 3.3 times higher risk of dying of breast cancer compared to women with 1–3 alleles (*P* = 0.001; HR, 3.30; 95% CI, 1.58–6.88). The results demonstrate the combined effect of variants in TTSPs and their related genes in breast cancer risk and patient outcome. Functional analysis of these variants will lead to further understanding of this gene family, which may improve individualized risk estimation and development of new strategies for treatment of breast cancer.

## Introduction

Breast cancer is the most common cancer among women in western countries. The known high risk susceptibility genes for breast cancer, e.g. *BRCA1*, *BRCA2*, *ATM*, and *PALB2*, are responsible for approximately 20% of the hereditary cases [1], but several unknown breast cancer–predisposing genetic factors still exist. Genetic risk factors with a low or moderate penetrance also affect the risk of sporadic breast cancers and may act together with environmental and lifestyle factors and with each other to enhance cancer predisposition and progression [2], [3].

Type II transmembrane serine proteases (TTSPs) degrade components of the extracellular matrix (ECM) [4], [5]. The 17 members of the human TTSP family have physiological and pathological roles in digestion, cardiac function, blood pressure regulation, hearing, iron metabolism, and epithelial homeostasis [4], [6]. In cancer, the TTSPs are related especially to tumor growth, invasion, and metastasis [7].

TTSPs are divided by their structures into subfamilies [4]. *TMPRSS1*, *TMPRSS2*, and *TMPRSS3* belong to the Hepsin/TMPRSS subfamily [4]. *TMPRSS1* is overexpressed in prostate and breast cancers, and its expression and localization have also been related to epithelial integrity [8], [9]. In terms of cancer-related risk, Pal and coworkers (2006) reported five *TMPRSS1* single nucleotide polymorphisms (SNPs) as associated with prostate cancer in men of European origin [10], although another study identified no associated variants [11]. The androgen-regulated *TMPRSS2*, however, is strongly associated with prostate cancer and forms a fusion gene with ETS transcription factor (TF) family members, that occurs in roughly half of prostate cancer cases [12], [13]. This fusion has been studied in ovarian cancer but not detected [14]. *TMPRSS3* is overexpressed in epithelial ovarian cancer and is a potential diagnostic marker and therapy target [15]–[18].

*TMPRSS11E/DESC1* belongs to the HAT/DESC subfamily of the TTSPs and is upregulated in tumors of different origins, including breast [19]. *DESC1* can convert pro-urokinase-type plasminogen activator (uPA, PLAU) to active uPA [19]. *uPA* belongs to the serine proteases and is an important factor in the plasminogen activation system associated with several cancers, including breast cancer and especially tumor invasion and metastasis [20]. In addition, uPA is suggested to be a suitable breast cancer biomarker when planning appropriate adjuvant therapy [21].

The third subfamily of the TTSPs is the matriptase subfamily. Matriptase/*ST14* is widely expressed in tissues rich in epithelial cells, such as breast, ovary, intestine, and prostate, and in tumors of epithelial origin derived from these tissues [22]–[24]. We have previously found that in the Eastern Finnish population, a variant in the *ST14* gene, rs704624, is associated with a poor patient outcome and low matriptase mRNA expression in breast cancer patients [25]. Moreover, negative/low matriptase protein expression is independently predictive of poor survival [25]. We also reported a genetic risk factor on *TMPRSS6*, coding matriptase-2, to be associated with elevated breast cancer risk and poor outcome [26], [27]. In addition, *TMPRSS6* is mutated in breast carcinomas [28]. *TMPRSS6* locates on chromosome 22q12-13 where an allelic imbalance has been observed in breast and colorectal cancers [29], [30]. *TMPRSS7*/Matriptase-3 is a recently found, evolutionary conserved TTSP expressed in brain, ovary, and testis [31]. No reports have described *TMPRSS7* in breast tissue or breast cancer.

Like *uPA*, *PRSS8* (Prostasin) is a serine protease activated by matriptase [32], [33]. Both uPA and PRSS8 are proteases involved in proteolytic cascades whereas hepatocyte growth factor (*HGF*) does not have proteolytic activity but instead is active in tumorigenesis, angiogenesis, and tissue regeneration via the HGF-Met pathway [34]. HGF is synthesized in pro-form, and like uPA and PRSS8, is processed to active form by the TTSP matriptase [35].

All of the genes investigated in this study are connected to the proteolytic activity that takes place through several cascades and leads to ECM degradation [4], [6]. The TTSPs are also involved in maintaining epithelial integrity [4], [6], [9], and in cancer, alterations in their function may destabilize tumor epithelia, and thus induce tumor invasion.

No published reports have addressed the association of most TTSP genetic variants with breast cancer. Here we evaluated the role of several TTSP variants and related genes in breast cancer patients. Motivated by our previous findings, we hypothesized that in addition to *ST14* [25] and *TMPRSS6* [26], [27], genes coding TTSPs and related genes exist as variants associated with breast cancer risk and patient outcome. To address this hypothesis, we genotyped tagging SNPs (tagSNPs) of five TTSP genes, *TMPRSS1*, *TMPRSS2*, *TMPRSS3*, *TMPRSS7*, and *TMPRSS11E*, two other serine proteases *uPA* and *PRSS8*, and *HGF*, and investigated their association with breast cancer risk and patient survival. We also tested whether the effect of the associated variants differs among the treatment groups and estimated the association of clinicopathological parameters with these variants.

## Materials and Methods

### DNA Samples

A sample set of 464 invasive breast cancer cases and 370 controls from the Kuopio Breast Cancer Project (KBCP) was available for genotyping (Table S1). The KBCP material consists of 497 prospective breast cancer cases and 458 controls from the province of Northern Savo in Eastern Finland. The cases were diagnosed at Kuopio University Hospital between April 1990 and December 1995, and the age- and long-term area-of-residence–matched controls were selected from the National Population Register during the same time period [25], [36], [37]. The maximum follow-up time of the patients was 20 years (February 2011). Genomic DNA was extracted from peripheral blood lymphocytes using standard procedures [38]. The KBCP is approved by the joint ethics committee of the University of Eastern Finland and the Kuopio University Hospital (written consents 1/1989 and 61/2010). Each patient gave informed written consent for participation in the study.

### SNP selection

TagSNPs for the *TMPRSS1*, *TMPRSS2*, *TMPRSS3*, *TMPRSS7*, *TMPRSS11E*, *PRSS8*, *uPA*, and *HGF* gene were selected using the HapMap Genome Browser release 2 (Phase 3, NCBI build 36, bdSNP b126) as of April 28, April 30, May 4, and May 5, 2009 (http://hapmap.ncbi.nlm.nih.gov/cgi-perl/gbrowse/hapmap3r2_B36/). TagSNPs for the regions chr19∶40218938-40253627 (*TMPRSS1*), chr21∶41748556-41811743 (*TMPRSS2*), chr21∶42661064->42693273 (*TMPRSS3*), chr3∶113225092-113298869 (*TMPRSS7*), chr4∶68980497-69061214 (*TMPRSS11E*), chr16∶31048796-31056113 (*PRSS8*), chr10∶75338443-75349712 (*uPA*) and chr7∶81153602-81253167 (*HGF*) were selected for the CEU (Utah residents with Northern and Western European ancestry from the CEPH collection) population using the Tagger multimarker algorithm with the r^2^ cutoff at 0.8 and minor allele frequency (MAF) cutoff at 0.05.

### SNP genotyping

Genotyping of 76/82 SNPs was done using MassARRAY (Sequenom Inc., San Diego, CA, USA) and iPLEX Gold (Sequenom Inc.) on 384-well plate format as previously described [39]. Duplicate analysis was done for 6.7% of KBCP samples for quality control. All primer sequences are available upon request. Six of the SNPs were genotyped using the 5′ nuclease assay (TaqMan) with the Mx3000P Real-Time PCR System (Stratagene, La Jolla, CA, USA) according to the manufacturer’s instructions. Primers and probes for *TMPRSS1* rs41523449 and *TMPRSS11E* rs2708699 were supplied by Applied Biosystems as Custom TaqMan SNP Genotyping Assays. *PRSS8* rs2855475, *TMPRSS3* rs2839506 and rs9325634, and *TMPRSS2* rs7275220 were supplied by Applied Biosystems as TaqMan Genotyping Assays. TaqMan genotyping was done as previously described [25]. TaqMan Genotyping Master Mix (Applied Biosystems) was used, as follows: 10 minutes at 95°C, 45–60 cycles of 15 seconds at 92°C, and 1 minute at 60°C. Duplicate genotypes were done for 4.2% of samples for quality control and the overall call rate was >95%. If the duplicate and its pair were discordant, the genotypes of the sample were discarded. Greater than 98% overall concordance was required for both iPLEX- and TaqMan-genotyped SNPs.

### Statistical analysis

Differences in SNP genotype frequencies between cases and controls were computed using the Armitage trend test (http://ihg.gsf.de/cgi-bin/hw/hwa1.pl) and logistic regression analysis. Concordance with Hardy–Weinberg equilibrium was calculated with the χ^2^ test. Association of the genotypes with clinicopathological variables was analyzed with the χ^2^ test, and the logistic regression analysis was used to evaluate the significance levels for the risks (odds ratios (ORs)) of the associated variables. Kaplan–Meier (log-rank test) analysis was used to calculate the breast cancer–specific survival (BCSS), and the multivariate survival analysis was performed using a Cox regression model. In all analyses *P*≤0.05 was considered significant. Statistical analyses were performed using SPSS v 19.0 (IBM SPSS statistics 19) and Haploview 4.2 [40]. *P* values were not corrected for multiple testing so as to avoid eliminating potentially important findings. Therefore, some of the results may need to be interpreted with caution and in addition be replicated in independent data sets. Genetic power estimation for the association studies was calculated using the Genetic Power Calculator, case-control for discrete traits at (http://pngu.mgh.harvard.edu/~purcell/gpc/) [41]. In the calculations, α was set as 0.05 and breast cancer prevalence as 1% [42]. The mean (0.23) of the observed MAFs of the genotyped SNPs in our sample set was used as the high-risk allele frequency. The allele frequencies were assumed to be equal for the risk SNP, and the marker SNP, and the D’ was set as 1 corresponding to perfect linkage disequilibrium (LD). The risk for the homozygous and heterozygous high-risk allele genotypes was assumed to be similar (1.2 or 1.5). *In silico* estimation for the SNP effects was done by using FastSNP [43] and F-SNP [44].

### Electrophoretic mobility shift assay

MCF7 cells were grown in minimum essential media containing 10% FBS, 1 mM sodium pyruvate, 1.5 g/l sodium bicarbonate, 1× NEAA, 2 mM L-glutamine, 0.01 mg/ml insulin, 100 U/ml penicillin, and 0.1 mg/ml streptomycin. For nuclear protein extraction, the cells were harvested in 1× PBS, and spun down for 5 minutes at 1000×*g* at 4°C. Pelleted cells were lysed in 4–5× volumes of lysis buffer [10 mM HEPES, 1.5 mM MgCl_2_, 10 mM KCl, 0.5 mM dithiothreitol, 0.5% (v/v) NP-40, protease inhibitors (Roche)] and incubated on ice for 5 minutes. Lysate was centrifuged for 1 minute at 12,000×*g* at 4°C and the supernatant discarded. The nuclear proteins were extracted in 2× volumes of extraction buffer [20 mM HEPES, 1.5 mM MgCl_2_, 420 mM NaCl, 0.2 mM EDTA, 0.5 mM dithiothreitol, 25% (v/v) glycerol, protease inhibitors (Roche)] for 30 minutes on ice, and vortexed a few times during incubation. Lysate was centrifuged for 1 minute at 12,000×*g* at 4°C, and the supernatant, containing nuclear proteins, was transferred to a fresh tube. Protein concentration was measured with the Bradford method using Coomassie brilliant blue (Merck, Darmstadt, Germany). Twenty-five micrograms of the protein extract was incubated for 40 minutes at 22°C with a 35 bp ^32^P-labelled DNA-oligomer corresponding to the T or C alleles of the rs12151195 (upper strand 5′-GCTCCTTCCTAAAATA**T/C**AGATGATCTACAAG-3′). DNA-oligomers were Klenow fill-in labeled. To prove the specific binding, 50× and 75× molar excesses of unlabeled oligomers were incubated with nuclear proteins for 10 minutes at 22°C prior to incubation with ^32^P-labeled oligomers. The complexes were separated at 22°C on 4% nondenaturing polyacrylamide gels using 0.25× tris-borate -EDTA buffer. The gels were dried and visualized using a phosphoimager (FLA3000; Fuji, Tokyo, Japan).

## Results

### SNPs in *TMPRSS3*, *TMPRSS7*, and *HGF* associate with breast cancer risk

Altogether, 82 SNPs in eight TTSPs and related genes were genotyped in a sample set of 464 invasive breast cancer cases and 370 controls (Table S2). *TMPRSS3* SNPs rs3814903 and rs11203200, *TMPRSS7* SNP rs1844925, and *HGF* SNP rs5745752 associated significantly with breast cancer risk (*P*_overall_ = 0.029, 0.008, 0.042, and 0.017, respectively) (Table 1). All SNPs were consistent with the Hardy–Weinberg equilibrium. According to the power calculations our sample set with 464 cases and 370 controls has 83% power to detect a risk allele that is in perfect LD with the marker allele and has a relative risk of 1.5.

**Table 1:** Breast cancer–associated SNP genotypes in invasive breast cancer cases and controls.

		Genotype counts				Homozygous^b^		Allele positivity^c^	
Gene and SNP	Genotype	Controls	Cases	*P* (Trend)^a^	Associated allele	*P*	OR (95% CI)	*P*	OR (95% CI)
*TMPRSS3*									
rs3814903				**0.029**	T	0.052	0.64 (0.41–1.01)	**0.038**	0.73 (0.54–0.98)
	GG	110	181						
	GT	167	208						
	TT	52	55						
rs11203200				**0.008**	A	0.109	6.00 (0.31–116.7)	**0.013**	1.692 (1.11–2.57)
	GG	319	372						
	GA	38	72						
	AA	0	3						
*TMPRSS7*				**0.042**	A	0.362	1.87 (0.48–7.28)	**0.046**	1.44 (1.01–2.05)
rs1844925	GG	271	339						
	GA	56	99						
	AA	3	7						
*HGF*									
rs5745752				**0.017**	A	**0.0087**	2.27 (1.22–4.25)	0.082	1.28 (0.97–1.70)
	GG	209	233						
	GA	131	171						
	AA	15	38						

a *P* (Trend); *P* value from the Armitage trend test for the overall association with invasive breast cancer risk.

b *P*, OR and CI for the homozygous allele carriers.

c *P*, OR and CI for the homozygous and heterozygous allele carriers.

Significant *P* values bolded.

### SNPs in *TMPRSS1*, *TMPRSS2*, *TMPRSS3* and *TMPRSS7* associate with breast cancer survival

**Figure 1:**
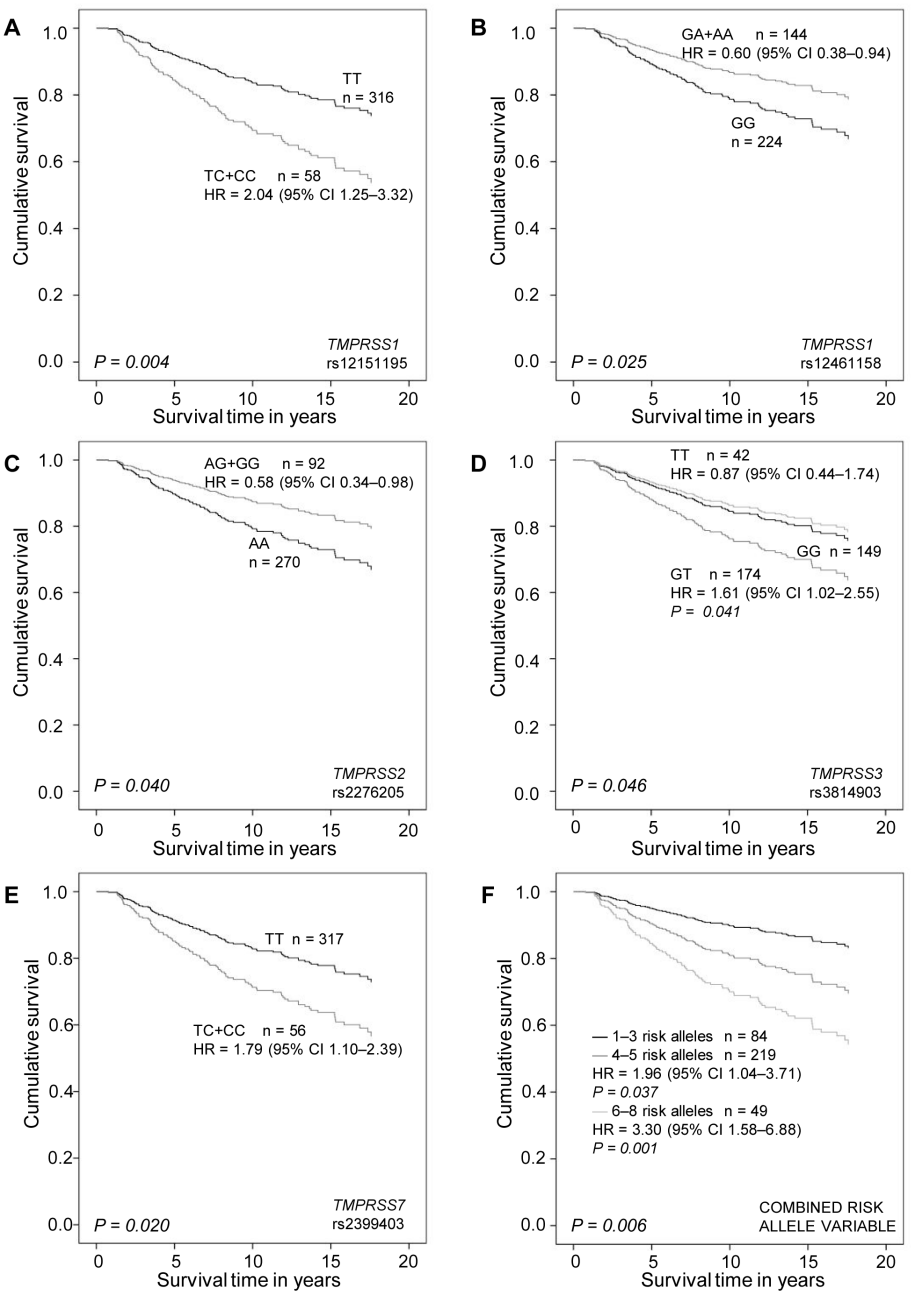
BCSS in multivariate analysis (Cox regression). A, rs12151195; B, rs12461158; C, rs2276205; D, rs3814903; E, rs2399403 genotypes; and F, combined risk allele variable. Age, tumor grade, histological type, tumor size, nodal status, ER status, and HER2 status were included in the multivariate analysis. *P*≤0.05 was considered significant.

In addition to breast cancer risk, we investigated variant association with patient survival. In the univariate analysis, *TMPRSS1* rs12151195 and rs12461158, *TMPRSS2* rs2070788 and rs2276205, *TMPRSS3* rs3814903, *TMPRSS7* rs2399403, *TMPRSS11E* rs35293564, *HGF* rs2040965, and *uPA* rs2227578 associated significantly with invasive breast cancer survival (*P* = 0.002, 0.05, 0.022, 0.05, 0.026, 0.007, 0.048, 0.035, and 0.021, respectively) (Table S3). *TMPRSS1* SNPs rs12151195 and rs12461158, *TMPRSS2* SNPs rs2276205, *TMPRSS3* SNP rs3814903, and *TMPRSS7* SNP rs2399403 remained significant in the multivariate analysis including age, tumor grade, histological type, tumor size, nodal status, estrogen receptor (ER) status, and HER2 status (*P* = 0.008, 0.025, 0.040, 0.046, and 0.047, respectively) (Table 2, Fig. 1). Only *TMPRSS3* rs3814903 associated with both the risk of breast cancer and survival. Of the clinical variables included in the survival analysis, tumor grade, nodal status, HER2 status, and histological type remained significant in all multivariate analyses. Association of the SNPs with the clinicopathological variables is shown as supplementary data (Table S4).

**Table 2:** BCSS in multivariate analysis (Cox regression) according to TTSP genotypes.

	Genotype	n	B (SE)	Wald	HR (95% CI)	*P* value
*TMPRSS1*						
rs12151195						**0.008** a
	TT	316		ref.		
	TC	57	0. 690 (0.252)	7.515	1.99 (1.22–3.26)	**0.006**
	CC	1	1.806 (1.056)	2.922	6.08 (0.77–48.2)	0.087
	TC+CC	58	0.712 (0.248)	8.226	2.04 (1.25–3.32)	**0.004**
rs12461158						
	GG	224		ref.		
	GA+AA	144	−0.519 (0.232)	5.001	0.60 (0.38–0.94)	**0.025**
*TMPRSS2*						
rs2276205						
	AA	270		ref.		
	AG+GG	92	−0.549 (0.267)	4.206	0.58 (0.34–0.98)	**0.040**
*TMPRSS3*						
rs3814903						**0.046** a
	GG	149		ref.		
	GT	174	0.478 (0.234)	4.166	1.61 (1.02–2.55)	**0.041**
	TT	42	−0.135 (0.351)	0.147	0.87 (0.44–1.74)	0.74
*TMPRSS7*						
rs2399403						**0.047** a
	TT	317		ref.		
	TC	52	0.645 (0.261)	6.101	1.91 (1.14–3.18)	**0.014**
	CC	4	0.140 (0.731)	0.037	1.15 (0.27–4.82)	0.84
	TC+CC	56	0.585 (0.250)	5.450	1.79 (1.10–2.93)	**0.020**
Combined risk allele variable						
						**0.006** a
	1–3 alleles	84		ref.		
	4–5 alleles	219	0.675 (0.324)	4.328	1.96 (1.04–3.71)	**0.037**
	6–8 alleles	49	1.193 (0.375)	10.13	3.30 (1.58–6.88)	**0.001**

a overall *P* value.

Significant *P* values bolded.

Note: Age, tumor grade, histological type, tumor size, nodal status, ER status, and HER2 status were included in the analysis.

Abbreviations: B (SE), coefficient with standard error, and ref., reference genotype.

### Increasing number of alleles significantly affects breast cancer risk and prognosis

To estimate the combined effect of the associating alleles, we assessed two new variables. We summed separately the number of alleles of four breast cancer risk-associating SNPs (rs3814903, rs11203200, rs1844925, and rs5745752) and five survival-associating SNPs (rs12151195, rs12461158, rs2276205, rs3814903, and rs2399403). In risk estimation women were divided into three groups carrying 0–2, 3, or 4–5 breast cancer risk alleles, since none of the cases or controls had six or more alleles (maximum eight). The risk of getting breast cancer was significantly higher with three risk alleles present (*P* = 0.003; OR, 1.70; 95% CI, 1.20–2.42, logistic regression analysis), and even higher with four to five alleles (*P* = 0.0001; OR, 2.34; 95% CI, 1.39–3.94, logistic regression analysis) compared to having 0–2 alleles.

In the multivariate survival analysis, patients with four or five risk alleles had a significantly poorer BCSS than those with one to three risk alleles (*P* = 0.037; HR, 1.96; 95% CI, 1.04–3.71) (Table 2, Fig. 1F). Moreover, the women with six to eight risk alleles had 3.3 times higher risk of dying of breast cancer compared with the women with one to three risk alleles (*P* = 0.001; HR, 3.30; 95% CI, 1.58–6.88) (Table 2, Fig. 1F). In the multivariate analysis including all five survival-related SNPs, only rs3814903 remained significant (overall *P* = 0.029, data not shown). All multivariate analyses included age, tumor grade, histological type, tumor size, nodal status, ER status, and HER2 status.

### Survival-associating SNPs in TTSPs affect patient outcome in different treatment groups

Overall survival (OS), BCSS, and recurrence-free survival (RFS) were assessed with a multivariate analysis according to the survival-associating SNPs and the combined risk allele variable in different treatment groups of the breast cancer patients. Among patients treated with radiation therapy *TMPRSS1* SNP rs12151195 and *TMPRSS3* SNP rs3814903 associated significantly with OS, BCSS, and RFS (*P* = 0.000001, 0.0003, and 0.013, and *P* = 0.016, 0.049, and 0.027, respectively) (Table 3). In addition, rs12461158 in *TMPRSS1* associated with OS and (*P* = 0.011), and *TMPRSS2* SNP rs2276205 with OS and BCSS (*P* = 0.019, and 0.020, respectively) (Table 3).

**Table 3:** Significant associations of the TTSP genotypes with survival among patients treated with radiation therapy.

			OS		BCSS		RFS	
Variable	Genotype	*n*	HR (95% CI)	*P* value^a^	HR (95% CI)	*P* value^a^	HR (95% CI)	*P* value^a^
*TMPRSS1*								
rs12151195								
	TT	187	ref.		ref.		ref.	
	TC+CC	41	3.07 (1.95–4.83)	0.000001	2.56 (1.60–4.52)	0.0003	1.84 (1.14–3.00)	0.013
rs12461158								
	AA	15	ref.					
	AG+GG	210	4.36 (1.59–11.9)	0.004				
*TMPRSS2*								
rs2276205								
	AA	167	ref.		ref.			
	AG+GG	56	0.58 (0.37–0.92)	0.019	0.49 (0.27–0.89)	0.020		
*TMPRSS3*								
rs3814903				0.016		0.049		
	GG	82	ref.		ref.		ref.	
	GT	120	1.69 (1.11–2.58)	0.014	1.66 (1.01–2.74)	0.046		
	TT	24	0.86 (0.45–1.67)	0.662	0.80 (0.35–1.82)	0.598		
	GT+TT	144					1.65 (1.06–2.59)	0.027
Combined risk allele variable				0.00001		0.00004		0.001
	1–3 alleles	50	ref.		ref.		ref.	
	4–5 alleles	138	1.71 (1.01–2.90)	0.045	1.91 (0.95–3.84)	0.069	1.74 (0.96–3.16)	0.067
	6–8 alleles	30	4.66 (2.42–8.98)	0.000004	5.35 (2.43–11.8)	0.00003	3.62 (1.78–7.38)	0.0004

a *P* value from multivariate Cox analysis.

Note: Age, tumor grade, histological type, tumor size, nodal status, ER status, HER2 status, hormonal treatment, and chemotherapy were included in the analysis.

Abbreviations: OS, overall survival; BCSS, breast cancer–specific survival; RFS, recurrence-free survival, and ref., reference genotype.

Among the patients receiving only radiation therapy *TMPRSS1* SNP rs12151195 and *TMPRSS3* rs3814903 associated with OS (*P* = 0.004, and *P* = 0.015, respectively) (Table 4).

**Table 4:** Significant associations of the TTSP genotypes with survival among patients treated with radiation therapy only.

			OS	
Variable	Genotype	*n*	HR (95% CI)	*P* value^a^
*TMPRSS1*				
rs12151195				
	TT	70	ref.	
	TC+CC	16	3.07 (1.43–6.56)	0.004
* TMPRSS3*				
rs3814903				
	GG+GT	75	ref.	
	TT	9	2.87 (1.23–6.67)	0.015
Combined risk allele variable				0.010
	1–3 alleles	20	ref.	
	4–5 alleles	50	1.80 (0.60–5.36)	0.295
	6–8 alleles	11	5.07 (1.54–16.6)	0.007

a *P* value from multivariate Cox analysis.

Note: Age, tumor grade, histological type, tumor size, nodal status, ER status, and HER2 status were included in the analysis.

Abbreviations: OS, overall survival, and ref., reference genotype.

In the group of patients treated with chemotherapy *TMPRSS3* rs3814903 G/T associated with BCSS (overall *P* = 0.025). Having rs3814903 major allele G carriers (n = 62) as a reference group, the TT homozygosity (n = 10) was significantly protective (*P* = 0.019; HR, 0.17; 95% CI, 0.04–0.75) when assessing BCSS. *TMPRSS3* rs3814903 associated also with RFS (*P* = 0.043).

An effect of the number of the survival-associating alleles was also seen in the different treatment groups. Women having more alleles had poorer OS and BCSS when treated with radiation therapy, compared with the women having fewer alleles (*P* = 0.00001 and 0.00004, respectively) (Table 3). Also, the RFS time was comparatively shorter among these women having more risk alleles (*P* = 0.001) (Table 3). The increasing number of risk alleles additionally affected the group treated with radiation therapy only: The OS was poorer among women carrying more than six risk alleles compared to those carrying five or fewer (*P* = 0.010) (Table 4). However, none of the these SNPs or the combined survival variant remained significant in the group of patients treated with hormone therapy or in the group receiving no treatment at all (data not shown). All multivariate analyses included age, tumor grade, histological type, tumor size, nodal status, ER status, and HER2 status.

### Nuclear proteins from breast cancer cells bind differentially to the rs12151195 T allele-harboring region

Because the rs12151195 T/C is a potential gene regulatory SNP, we tested whether the C/T difference influences the binding of nuclear proteins to this gene region. To that end electrophoretic mobility shift assay with nuclear proteins from human breast cancer cells was performed. Interestingly, the common allele T-harboring ds-oligomer showed formation of a high molecular mass nuclear protein–DNA complex that was not evident with the rare C allele (Figure S1). However, the identity of the bound nuclear protein remains to be elucidated.

## Discussion

Proteolytic enzymes like the TTSPs associate with tumor invasion and metastasis in cancer. In this study, we genotyped 82 tagSNPs from seven serine protease genes and *HGF* and evaluated their role in breast cancer risk and survival. We found both risk- and survival-associating variants in five genes: *TMPRSS1*, *TMPRSS2*, *TMPRSS3*, *TMPRSS7*, and *HGF*. More important, we found that the more breast cancer risk-associating or survival-associating variants from this gene family the women had, the higher the risk of developing breast cancer or dying of it. Some of the survival-associating variants and especially the combined survival variants maintained their impairing effect also when treatment regimens were included to the analysis. These results suggest that genetic alterations disturb the function of these genes and proteins to cause enhanced proteolysis and epithelial disorder, possibly leading to cancer onset and progression.

Four variants in this study associated with breast cancer risk. The strongest single marker association was with *TMPRSS3* intronic variant rs11203200; the minor allele carriers had a greater than 1.6-fold risk of developing breast cancer compared with the major allele homozygotes. The minor allele homozygotes were very rare; only three patients in our sample set carried it in both chromosomes. In the *in silico* analysis, this variant seems to have a possible effect as an intronic enhancer, and the minor allele may destroy the binding site of TF lyf-1. In addition, a 5′ untranslated region (UTR) variant is in strong LD with rs11203200 (1000 Genomes Project) [45]. The 5′UTR contains regulatory elements, and its variation thus may affect gene expression. Although no studies have previously evaluated the role of *TMPRSS3* in breast cancer, it is overexpressed in epithelial ovarian cancer, and is a potential diagnostic marker and therapy target [15]–[18]. Another breast cancer risk-associating *TMPRSS3* variant in our study was rs3814903. Whether these variants are responsible for the possible changes in gene expression on the mRNA or protein level in breast cancer remains to be elucidated.

*TMPRSS7* (matriptase-3) is very rarely studied with only one publication by Szabo and colleagues (2005) [31] and has no known role in any cancer, but we found here that rs1844925 associated with breast cancer risk. It is an intronic variant at the start site of the gene near a missense variant rs11929695 that is in LD with rs1844925 (r^2^ = 0.759), and changes an amino acid leading to possible effects (1000 Genomes Project) [45].

HGF works via its cognate receptor, Met, with several roles in signaling in different pathways. It can be linked to cancer progression [34], but there are no published associations with breast cancer risk. We found that the women homozygous for the minor allele of *HGF* SNP rs5745752 had a higher risk of breast cancer whereas the major allele was significantly protective. In addition, the minor alleles of three *HGF* variants associated with the clinical variables of higher tumor grade, positive nodal status, bigger tumor size, negative ER and PR status, and overall higher tumor stage. These associations may support the role of *HGF* in many actions of cancer progression.

Our results strongly indicate that an increased number of risk alleles enhances the risk of breast cancer. Women who had four to five risk alleles had 2.3-fold higher risk of breast cancer compared with women carrying up to two risk alleles, and the overall risk with the combined risk allele variable was higher than with any of the SNPs on their own. This result, even with the small number of genes involved, supports the polygenic risk model as suggested by recently published iCOGS (Collaborative Oncological Gene-environment Study) studies [46]. Here, the associated variants are all non-coding and do not affect the structure of the proteins translated from these genes. The variants may, however, affect the transcriptional regulation of these genes and thus disrupt signaling in and among cell types. In the case of tagSNPs, the effective or functional variant may also be nearby in the same LD block.

In addition to breast cancer risk estimation, we studied the association of the serine protease genetic variants with BCSS and survival in different treatment groups. We found five breast cancer survival–associated SNPs in four TTSP genes, and also evidence about poor response to radiotherapy due to variants. The strongest association was with the minor allele of *TMPRSS1* rs12151195, which was a marker of poor prognosis and associated with PR-negative tumors. In addition, the minor allele carriers of the variant rs12151195 had poorer OS, BCSS, and RFS among patients given radiotherapy. rs12151195 is located after the 3′UTR of *TMPRSS1*, but its potential regulatory function is not known. Interestingly, the T/C difference in the rs12151195 influences the binding of nuclear protein(s) to the gene region; however, because the identity of the putative TF binding to the gene region is currently unclear (*in silico* searches failed to predict a strong candidate TF), it is difficult to judge the possible regulatory effect of the SNP. According to the 1000 Genomes Project data from Finns, more than 20 variants are present in a 25-kb area with LD (r^2^) ≥0.75 in both directions from rs12151195 [45]. Further studies taking these variables into account are needed. In the same gene, *TMPRSS1*, the minor allele of rs12461158 associated with better survival, as well as with negative HER2 status. In two other studies, *TMPRSS1* gene variants have been found to associate with prostate cancer susceptibility but not with the prognosis [10], [47]. However, the genotyped variants were not the same as in our study. *TMPRSS1* expression in breast cancer is enhanced on the protein level, assessed with immunohistochemistry [8]. In that study, the knockdown of *TMPRSS1* in breast cancer cells with high *TMPRSS1* expression led to a decreased invasion in a Matrigel invasion assay, suggesting it to have a role in tumor invasion [8]. Most of the studies concerning *TMPRSS1* and *TMPRSS2* are done with prostate cancer, and very little is known about their role in breast cancer.

Our results show that the *TMPRSS2* rs2276205 minor allele is associated with better survival in breast cancer patients; thus, the major allele impairs the prognosis. Interestingly, an *in silico* analysis showed that the minor allele possibly disrupts the binding site of GATA-1, which is present with the major allele. In addition, the minor allele associated with tumor PR positivity, which may be connected with survival via treatment. Silencing of *TMPRSS2* causes sensitivity to tamoxifen, one of the most widely used drugs in treating breast cancer [48]. Moreover, *TMPRSS2* is androgen-regulated and forms a fusion gene with ETS TFs in prostate cancer [13]. Whether the fusion gene occurs in breast cancer is not known. Interestingly, androgens and the androgen receptor affect breast cancer risk and prognosis, although the data are somewhat complicated [49].

In our study, the only SNP associated with both breast cancer risk and survival was *TMPRSS3* rs3814903. Surprisingly, the patients who were heterozygous for *TMPRSS3* rs3814903 had poorer survival than those who were homozygous. The SNP sits approximately 1100 bp upstream from the gene *TMPRSS3* and may thus affect regulation of gene expression. The *in silico* analysis suggested rs3814903 to associate with splicing regulation and detected the minor allele to create a binding motif for splicing factor SRp55 that is absent with the major allele. In addition, in the same analysis, the major allele creates a binding site for the splicing factor SF2/ASF, also known as SRSF1. SRSF1 is a reported proto-oncogene and involved in mammary epithelial transformation via its overexpression and by splicing regulation of Bcl-2 family tumor suppressors [50], [51].

Interestingly, *TMPRSS7*, encoding matriptase-3, was the only gene having SNPs associated with both breast cancer risk and prognosis. Our results show that the *TMPRSS7* intronic variant rs2399403 associated with breast cancer survival. Compared with those who were homozygous for the major allele, the minor allele carriers had significantly poorer survival. Moreover, in the group of patients with no adjuvant therapy, the rare allele carriers of rs2399403 had significantly poorer BCSS, and in addition, their RFS was slightly significantly shorter (no data shown). Previously, Szabo and colleagues (2005) identified matriptase-3 as a functional serine protease [31]; therefore, it might affect tumor invasiveness. More important, *TMPRSS7* belongs to the same TTSP subfamily as matriptase and matriptase-2 that we have found to be associated with breast cancer, which makes it of interest for further study. [25]–[27].

As with the breast cancer risk–associating SNPs, we combined the survival-associated SNPs into a new variable and estimated its association with prognosis. The risk of dying of breast cancer was 3.3-fold higher among women who had six to eight risk alleles compared with women with one to three alleles. The effect of an increasing number of risk alleles was also seen in radiation therapy group. However, this was not the case in the group of patients with no adjuvant treatment (no data shown). Therefore, although some of the TTSP SNPs were significantly associated with survival in the whole study population, this association might reflect their prediction of response to cancer therapies rather than their role as prognostic markers. The studied SNPs might associate with patient outcome by affecting metabolic pathways or response to cytotoxic treatments. To our knowledge, though, no *in vitro* or clinical data address these specific SNPs and their potential association with the efficacy of radiotherapy or chemotherapy.

In summary, the results of this study suggest that these genetic variants should be evaluated as an overall pattern instead of as single markers when assessing patient cancer risk, prognosis, and treatment. If the breast cancer risk- and survival-associating SNPs lead to changes in gene function or expression levels, they may affect on tumor invasion, metastasis, and response to treatment as a whole protein family. The effects of different TTSPs on the enhancement of the tumor invasion are not necessarily parallel, but we can hypothesize that one overexpressed protein enhances ECM degradation and that underexpression of another protein destabilizes epithelial integrity [9], [34]. Small sequence changes can affect TF binding or epigenetic regulation and thus lead to changes in gene expression. However, this idea needs to be studied *in vivo* in functional studies of these associated variants to confirm the current results.

## Supporting Information

Figure S1 **Differential binding of nuclear proteins from breast cancer cells to oligomers corresponding to rs12151195 alleles.** Electrophoretic mobility shift assay was performed as described in the materials and methods. The upper arrow indicates the position of differential DNA–protein complex formation. The lower arrow indicates the free probe. Lanes 1 and 2: oligomers corresponding to rs12151195 C and T alleles without proteins; lanes 3 and 4: oligomers corresponding to rs12151195 C and T alleles with 25 µg of MCF7 breast cancer cell nuclear proteins; lanes 5 and 6: oligomers corresponding to rs12151195 C and T alleles with 25 µg of MCF7 cell nuclear proteins and with 50× molar excess of unlabeled oligomers; and lanes 7 and 8: oligomers corresponding to rs12151195 C and T alleles with 25 µg of MCF7 cell nuclear proteins and with 75× molar excess of unlabeled oligomers.
(TIF)

Table S1 **Clinicopathological characteristics of all patients (invasive cases).**
(DOCX)

Table S2 **SNP genotype counts in invasive breast cancer cases, (including metastatic cases) and controls.**
(DOCX)

Table S3 **Breast cancer survival in univariate analysis (Kaplan–Meier) at the latest follow-up data point.**
(DOCX)

Table S4 **Significant associations of gene variants with clinical variables.**
(DOCX)
