# Supplementary figures and images for: Type II Transmembrane Serine Protease Gene Variants Associate with Breast Cancer
Source: PLoS One. 2014 Jul 16;9(7):e102519. doi: 10.1371/journal.pone.0102519 (PMC4100901; doi:10.1371/journal.pone.0102519)

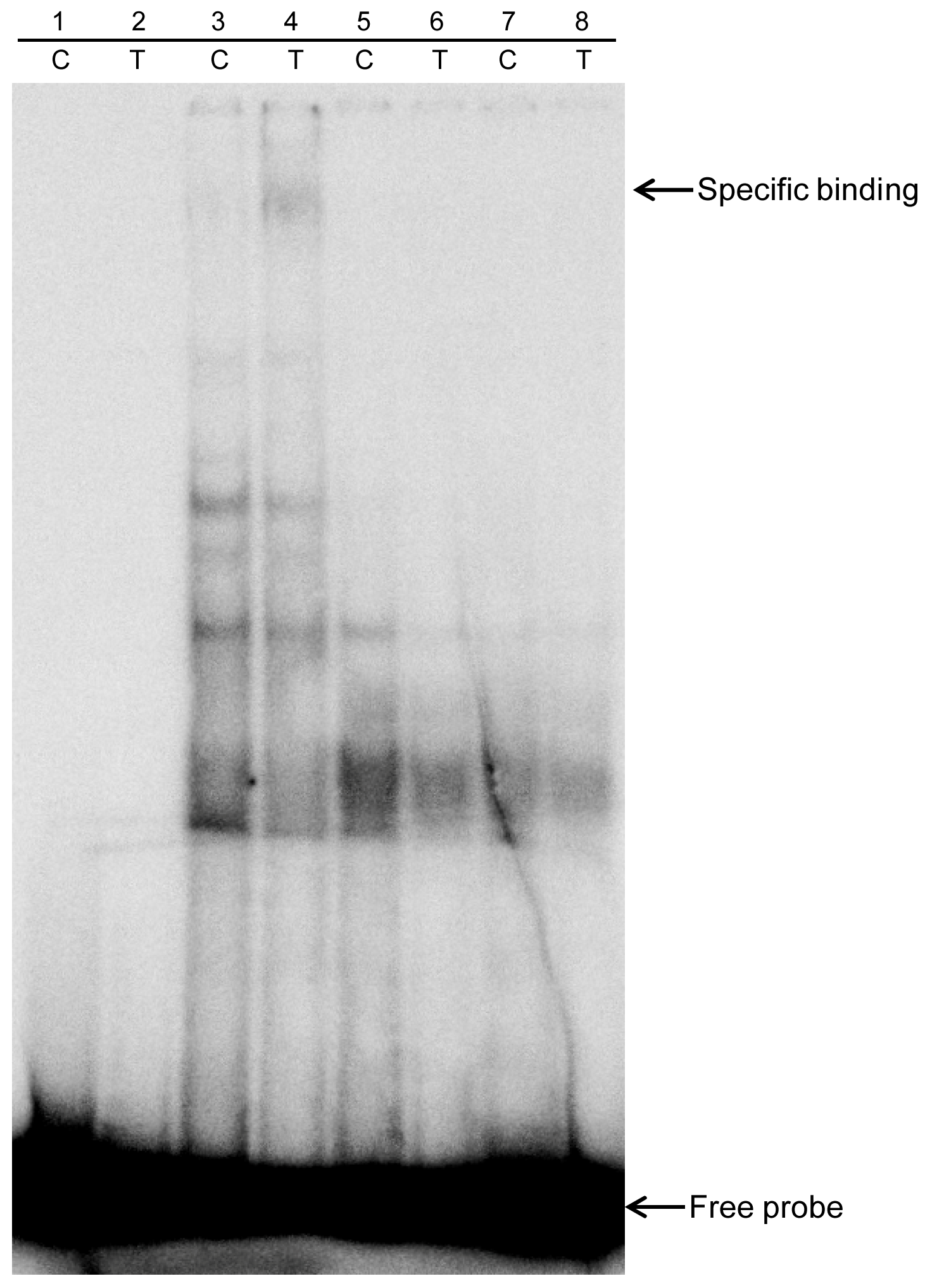

Supplement: Figure S1 — Differential binding of nuclear proteins from breast cancer cells to oligomers corresponding to rs12151195 alleles. Electrophoretic mobility shift assay was performed as described in the materials and methods. The upper arrow indicates the position of differential DNA–protein complex formation. The lower arrow indicates the free probe. Lanes 1 and 2: oligomers corresponding to rs12151195 C and T alleles without proteins; lanes 3 and 4: oligomers corresponding to rs12151195 C and T alleles with 25 µg of MCF7 breast cancer cell nuclear proteins; lanes 5 and 6: oligomers corresponding to rs12151195 C and T alleles with 25 µg of MCF7 cell nuclear proteins and with 50× molar excess of unlabeled oligomers; and lanes 7 and 8: oligomers corresponding to rs12151195 C and T alleles with 25 µg of MCF7 cell nuclear proteins and with 75× molar excess of unlabeled oligomers. (TIF) [file pone.0102519.s001.tif]
