# Supplementary material for: Type II Transmembrane Serine Protease Gene Variants Associate with Breast Cancer
Source: PLoS One. 2014 Jul 16;9(7):e102519. doi: 10.1371/journal.pone.0102519 (PMC4100901; doi:10.1371/journal.pone.0102519)
Supplement: Table S1 — Clinicopathological characteristics of all patients (invasive cases). (DOCX) [file pone.0102519.s002.docx]

**Supplemental Table S1.**

| **Characteristic** | ***n*** | **%** |
| --- | --- | --- |
| Number of patients | 464 | 100 |
| Tumor grade |  |  |
| I | 116 | 25.0 |
| II | 203 | 43.8 |
| III | 117 | 25.2 |
| NA | 28 | 6.0 |
| Histological type |  |  |
| Ductal | 280 | 60.3 |
| Lobular | 81 | 17.5 |
| Other | 77 | 16.6 |
| NA | 26 | 5.6 |
| Tumor size |  |  |
| T1 | 223 | 48.1 |
| T2 | 174 | 37.5 |
| T3 | 24 | 5.2 |
| T4 | 17 | 3.7 |
| NA | 26 | 5.5 |
| Nodal status |  |  |
| Negative | 254 | 54.7 |
| Positive | 175 | 37.7 |
| Unknown | 9 | 1.9 |
| NA | 26 | 5.7 |
| ER status |  |  |
| Negative | 96 | 20.7 |
| Positive | 328 | 70.7 |
| Unknown | 14 | 3.0 |
| NA | 26 | 5.6 |
| Stage |  |  |
| 0 | 1 | 0.2 |
| I | 167 | 36.0 |
| II | 209 | 45.0 |
| III | 35 | 7.5 |
| IV | 16 | 3.4 |
| Unknown | 10 | 2.2 |
| NA | 26 | 5.7 |
| HER2 status |  |  |
| Negative | 356 | 76.7 |
| Positive | 52 | 11.2 |
| NA | 56 | 12.1 |
| Cause of death |  |  |
| Alive | 200 | 43.1 |
| Breast cancer | 131 | 28.2 |
| Other | 133 | 28.7 |

Abbreviations: NA, data not available
