# Supplementary material for: Type II Transmembrane Serine Protease Gene Variants Associate with Breast Cancer
Source: PLoS One. 2014 Jul 16;9(7):e102519. doi: 10.1371/journal.pone.0102519 (PMC4100901; doi:10.1371/journal.pone.0102519)
Supplement: Table S2 — SNP genotype counts in invasive breast cancer cases, (including metastatic cases) and controls. (DOCX) [file pone.0102519.s003.docx]

**Supplemental Table S2.**

|  | **MAF** | |  | **Genotype counts** | |  |
| --- | --- | --- | --- | --- | --- | --- |
| **Gene and SNP** | **Controls** | **Cases** | **Genotype** | **Controls** | **Cases** | ***P* value**^a^ |
| *PRSS8* |  |  |  |  |  |  |
| rs2855475 | 0.37 | 0.36 | AA | 145 | 176 | 0.597 |
|  |  |  | AG | 162 | 219 |  |
|  |  |  | GG | 54 | 52 |  |
|  |  |  | Total | 361 | 447 |  |
|  |  |  |  |  |  |  |
| rs11645814 | 0.34 | 0.31 | GG | 137 | 215 | 0.109 |
|  |  |  | GA | 151 | 184 |  |
|  |  |  | AA | 36 | 43 |  |
|  |  |  | Total | 324 | 442 |  |
| *uPA* |  |  |  |  |  |  |
| rs2227578 | 0.47 | 0.48 | GG | 106 | 118 | 0.501 |
|  |  |  | GA | 160 | 224 |  |
|  |  |  | AA | 83 | 104 |  |
|  |  |  | Total | 349 | 446 |  |
|  |  |  |  |  |  |  |
| rs2459449 | 0.39 | 0.37 | CC | 128 | 175 | 0.427 |
|  |  |  | CT | 167 | 211 |  |
|  |  |  | TT | 52 | 60 |  |
|  |  |  | Total | 347 | 446 |  |
| *TMPRSS1* |  |  |  |  |  |  |
| rs870379 | 0.32 | 0.32 | TT | 158 | 209 | 0.798 |
|  |  |  | TC | 155 | 190 |  |
|  |  |  | CC | 34 | 45 |  |
|  |  |  | Total | 347 | 444 |  |
|  |  |  |  |  |  |  |
| rs1688029 | 0.28 | 0.30 | AA | 188 | 222 | 0.535 |
|  |  |  | AG | 147 | 191 |  |
|  |  |  | GG | 30 | 39 |  |
|  |  |  | Total | 365 | 452 |  |
|  |  |  |  |  |  |  |
| rs2305747 | 0.21 | 0.24 | TT | 215 | 261 | 0.161 |
|  |  |  | TC | 100 | 160 |  |
|  |  |  | CC | 19 | 26 |  |
|  |  |  | Total | 334 | 447 |  |
|  |  |  |  |  |  |  |
| rs8107142 | 0.13 | 0.11 | GG | 265 | 356 | 0.146 |
|  |  |  | GA | 83 | 87 |  |
|  |  |  | AA | 5 | 5 |  |
|  |  |  | Total | 353 | 448 |  |
|  |  |  |  |  |  |  |
| rs12151195 | 0.06 | 0.08 | TT | 321 | 385 | 0.065 |
|  |  |  | TC | 36 | 66 |  |
|  |  |  | CC | 2 | 3 |  |
|  |  |  | Total | 359 | 454 |  |
|  |  |  |  |  |  |  |
| rs12461158 | 0.22 | 0.23 | GG | 216 | 267 | 0.678 |
|  |  |  | GA | 123 | 153 |  |
|  |  |  | AA | 18 | 27 |  |
|  |  |  | Total | 357 | 447 |  |
|  |  |  |  |  |  |  |
| rs41523449 | 0.15 | 0.16 | CC | 258 | 321 | 0.601 |
|  |  |  | CT | 89 | 106 |  |
|  |  |  | TT | 8 | 17 |  |
|  |  |  | Total | 355 | 444 |  |
| *TMPRSS2* |  |  |  |  |  |  |
| rs462471 | 0.26 | 0.29 | GG | 183 | 222 | 0.227 |
|  |  |  | GA | 143 | 191 |  |
|  |  |  | AA | 20 | 35 |  |
|  |  |  | Total | 346 | 448 |  |
|  |  |  |  |  |  |  |
| rs468397 | 0.52 | 0.50 | GG | 82 | 113 | 0.525 |
|  |  |  | GC | 178 | 218 |  |
|  |  |  | CC | 95 | 115 |  |
|  |  |  | Total | 355 | 446 |  |
|  |  |  |  |  |  |  |
| rs468811 | 0.23 | 0.28 | GG | 208 | 236 | 0.054 |
|  |  |  | GA | 121 | 180 |  |
|  |  |  | AA | 21 | 34 |  |
|  |  |  | Total | 350 | 450 |  |
|  |  |  |  |  |  |  |
| rs734056 | 0.39 | 0.38 | CC | 126 | 172 | 0.683 |
|  |  |  | CA | 162 | 209 |  |
|  |  |  | AA | 53 | 67 |  |
|  |  |  | Total | 341 | 448 |  |
|  |  |  |  |  |  |  |
| rs2070788 | 0.38 | 0.37 | TT | 140 | 176 | 0.755 |
|  |  |  | TC | 168 | 212 |  |
|  |  |  | CC | 53 | 61 |  |
|  |  |  | Total | 361 | 449 |  |
|  |  |  |  |  |  |  |
| rs2070790 | 0.24 | 0.23 | CC | 203 | 267 | 0.694 |
|  |  |  | CG | 126 | 158 |  |
|  |  |  | GG | 20 | 24 |  |
|  |  |  | Total | 349 | 449 |  |
|  |  |  |  |  |  |  |
| rs2187238 | 0.13 | 0.14 | TT | 246 | 331 | 0.788 |
|  |  |  | TC | 79 | 102 |  |
|  |  |  | CC | 5 | 11 |  |
|  |  |  | Total | 330 | 444 |  |
|  |  |  |  |  |  |  |
| rs2276205 | 0.17 | 0.14 | AA | 231 | 326 | 0.177 |
|  |  |  | AG | 98 | 103 |  |
|  |  |  | GG | 8 | 12 |  |
|  |  |  | Total | 337 | 441 |  |
|  |  |  |  |  |  |  |
| rs2838039 | 0.45 | 0.44 | TT | 108 | 146 | 0.684 |
|  |  |  | TC | 182 | 209 |  |
|  |  |  | CC | 75 | 96 |  |
|  |  |  | Total | 365 | 451 |  |
|  |  |  |  |  |  |  |
| rs2838042^b^ | 0.32 | 0.33 | TT | 221 | 210 | 0.718 |
|  |  |  | TC | 198 | 195 |  |
|  |  |  | CC | 50 | 51 |  |
|  |  |  | Total | 469 | 456 |  |
|  |  |  |  |  |  |  |
| rs3761373 | 0.10 | 0.09 | CC | 293 | 375 | 0.435 |
|  |  |  | CT | 65 | 73 |  |
|  |  |  | TT | 5 | 5 |  |
|  |  |  | Total | 363 | 453 |  |
|  |  |  |  |  |  |  |
| rs3761374 | 0.23 | 0.24 | TT | 193 | 249 | 0.481 |
|  |  |  | TC | 123 | 172 |  |
|  |  |  | CC | 14 | 22 |  |
|  |  |  | Total | 330 | 443 |  |
|  |  |  |  |  |  |  |
| rs3787950 | 0.02 | 0.02 | TT | 344 | 430 | 0.933 |
|  |  |  | TC | 12 | 17 |  |
|  |  |  | CC | 1 | 0 |  |
|  |  |  | Total | 357 | 447 |  |
|  |  |  |  |  |  |  |
| rs6517672 | 0.26 | 0.25 | CC | 147 | 237 | 0.718 |
|  |  |  | CT | 104 | 154 |  |
|  |  |  | TT | 19 | 30 |  |
|  |  |  | Total | 270 | 421 |  |
|  |  |  |  |  |  |  |
| rs7275220 | 0.14 | 0.18 | AA | 248 | 307 | 0.063 |
|  |  |  | AG | 87 | 119 |  |
|  |  |  | GG | 5 | 20 |  |
|  |  |  | Total | 340 | 446 |  |
|  |  |  |  |  |  |  |
| rs8127664 | 0.26 | 0.29 | CC | 169 | 220 | 0.222 |
|  |  |  | CT | 131 | 194 |  |
|  |  |  | TT | 17 | 31 |  |
|  |  |  | Total | 317 | 445 |  |
|  |  |  |  |  |  |  |
| rs8127674 | 0.36 | 0.37 | AA | 150 | 177 | 0.885 |
|  |  |  | AG | 163 | 214 |  |
|  |  |  | GG | 51 | 58 |  |
|  |  |  | Total | 364 | 449 |  |
|  |  |  |  |  |  |  |
| rs8133719 | 0.49 | 0.49 | TT | 95 | 120 | 0.926 |
|  |  |  | TG | 179 | 219 |  |
|  |  |  | GG | 87 | 108 |  |
|  |  |  | Total | 361 | 447 |  |
|  |  |  |  |  |  |  |
| rs9984523 | 0.11 | 0.12 | CC | 278 | 352 | 0.943 |
|  |  |  | CT | 71 | 92 |  |
|  |  |  | TT | 5 | 6 |  |
|  |  |  | Total | 354 | 450 |  |
|  |  |  |  |  |  |  |
| rs9985159 | 0.38 | 0.39 | CC | 135 | 171 | 0.646 |
|  |  |  | CT | 173 | 207 |  |
|  |  |  | TT | 47 | 70 |  |
|  |  |  | Total | 355 | 448 |  |
| *TMPRSS3* |  |  |  |  |  |  |
| rs186531 | 0.14 | 0.11 | CC | 268 | 357 | 0.104 |
|  |  |  | CT | 90 | 91 |  |
|  |  |  | TT | 5 | 5 |  |
|  |  |  | Total | 363 | 453 |  |
|  |  |  |  |  |  |  |
| rs225310 | 0.41 | 0.38 | GG | 125 | 181 | 0.247 |
|  |  |  | GT | 168 | 197 |  |
|  |  |  | TT | 60 | 72 |  |
|  |  |  | Total | 353 | 450 |  |
|  |  |  |  |  |  |  |
| rs225313 | 0.21 | 0.23 | AA | 202 | 265 | 0.314 |
|  |  |  | AT | 114 | 152 |  |
|  |  |  | TT | 12 | 27 |  |
|  |  |  | Total | 328 | 444 |  |
|  |  |  |  |  |  |  |
| rs225433 | 0.17 | 0.18 | CC | 242 | 302 | 0.772 |
|  |  |  | CG | 97 | 137 |  |
|  |  |  | GG | 13 | 13 |  |
|  |  |  | Total | 352 | 452 |  |
|  |  |  |  |  |  |  |
| rs462149^b^ | 0.15 | 0.13 | GG | 334 | 342 | 0.190 |
|  |  |  | GA | 131 | 110 |  |
|  |  |  | AA | 6 | 5 |  |
|  |  |  | Total | 471 | 457 |  |
|  |  |  |  |  |  |  |
| rs1078272 | 0.36 | 0.34 | TT | 146 | 190 | 0.277 |
|  |  |  | TA | 166 | 209 |  |
|  |  |  | AA | 48 | 46 |  |
|  |  |  | Total | 360 | 445 |  |
|  |  |  |  |  |  |  |
| rs2251362 | 0.09 | 0.09 | GG | 273 | 368 | 0.897 |
|  |  |  | GA | 57 | 73 |  |
|  |  |  | AA | 1 | 4 |  |
|  |  |  | Total | 331 | 445 |  |
|  |  |  |  |  |  |  |
| rs2839489 | 0.25 | 0.26 | TT | 195 | 243 | 0.653 |
|  |  |  | TG | 140 | 172 |  |
|  |  |  | GG | 18 | 29 |  |
|  |  |  | Total | 353 | 444 |  |
|  |  |  |  |  |  |  |
| rs2839493 | 0.08 | 0.08 | GG | 305 | 381 | 0.781 |
|  |  |  | GA | 57 | 66 |  |
|  |  |  | AA | 2 | 3 |  |
|  |  |  | Total | 364 | 450 |  |
|  |  |  |  |  |  |  |
| rs2839501 | 0.28 | 0.26 | CC | 170 | 245 | 0.409 |
|  |  |  | CT | 139 | 169 |  |
|  |  |  | TT | 22 | 30 |  |
|  |  |  | Total | 331 | 444 |  |
|  |  |  |  |  |  |  |
| rs2839502 | 0.24 | 0.22 | GG | 205 | 270 | 0.318 |
|  |  |  | GA | 138 | 154 |  |
|  |  |  | AA | 18 | 21 |  |
|  |  |  | Total | 361 | 445 |  |
|  |  |  |  |  |  |  |
| rs2839506 | 0.05 | 0.06 | AA | 312 | 396 | 0.633 |
|  |  |  | AG | 36 | 51 |  |
|  |  |  | GG | 0 | 0 |  |
|  |  |  | Total | 348 | 447 |  |
|  |  |  |  |  |  |  |
| rs3814899 | 0.17 | 0.18 | GG | 248 | 300 | 0.594 |
|  |  |  | GC | 100 | 133 |  |
|  |  |  | CC | 11 | 14 |  |
|  |  |  | Total | 359 | 447 |  |
|  |  |  |  |  |  |  |
| rs3814903 | 0.41 | 0.36 | GG | 110 | 181 | **0.029** |
|  |  |  | GT | 167 | 208 |  |
|  |  |  | TT | 52 | 55 |  |
|  |  |  | Total | 329 | 444 |  |
|  |  |  |  |  |  |  |
| rs3829962 | 0.07 | 0.06 | CC | 313 | 398 | 0.220 |
|  |  |  | CT | 52 | 51 |  |
|  |  |  | TT | 1 | 1 |  |
|  |  |  | Total | 366 | 450 |  |
|  |  |  |  |  |  |  |
| rs7277003 | 0.08 | 0.07 | TT | 313 | 394 | 0.515 |
|  |  |  | TC | 47 | 57 |  |
|  |  |  | CC | 5 | 3 |  |
|  |  |  | Total | 365 | 454 |  |
|  |  |  |  |  |  |  |
| rs9325634 | 0.37 | 0.38 | AA | 141 | 173 | 0.791 |
|  |  |  | AG | 147 | 205 |  |
|  |  |  | GG | 54 | 66 |  |
|  |  |  | Total | 342 | 444 |  |
|  |  |  |  |  |  |  |
| rs9981459 | 0.10 | 0.12 | CC | 291 | 347 | 0.176 |
|  |  |  | CG | 71 | 92 |  |
|  |  |  | GG | 2 | 10 |  |
|  |  |  | Total | 364 | 449 |  |
|  |  |  |  |  |  |  |
| rs11203200 | 0.05 | 0.09 | GG | 319 | 372 | **0.008** |
|  |  |  | GA | 38 | 72 |  |
|  |  |  | AA | 0 | 3 |  |
|  |  |  | Total | 357 | 447 |  |
|  |  |  |  |  |  |  |
| rs13047838 | 0.17 | 0.19 | CC | 237 | 297 | 0.481 |
|  |  |  | CT | 99 | 134 |  |
|  |  |  | TT | 10 | 16 |  |
|  |  |  | Total | 346 | 447 |  |
| *TMPRSS7* |  |  |  |  |  |  |
| rs168150 | 0.36 | 0.35 | TT | 130 | 184 | 0.724 |
|  |  |  | TC | 133 | 189 |  |
|  |  |  | TT | 44 | 56 |  |
|  |  |  | Total | 307 | 429 |  |
|  |  |  |  |  |  |  |
| rs340146 | 0.03 | 0.04 | CC | 332 | 416 | 0.322 |
|  |  |  | CT | 21 | 33 |  |
|  |  |  | TT | 0 | 1 |  |
|  |  |  | Total | 353 | 450 |  |
|  |  |  |  |  |  |  |
| rs340151 | 0.06 | 0.04 | TT | 304 | 405 | 0.127 |
|  |  |  | TA | 39 | 38 |  |
|  |  |  | AA | 1 | 0 |  |
|  |  |  | Total | 344 | 443 |  |
|  |  |  |  |  |  |  |
| rs732686 | 0.17 | 0.16 | TT | 230 | 311 | 0.860 |
|  |  |  | TC | 90 | 121 |  |
|  |  |  | CC | 10 | 12 |  |
|  |  |  | Total | 330 | 444 |  |
|  |  |  |  |  |  |  |
| rs774770 | 0.01 | 0.01 | GG | 351 | 443 | 0.984 |
|  |  |  | GA | 8 | 10 |  |
|  |  |  | AA | 0 | 0 |  |
|  |  |  | Total | 359 | 453 |  |
|  |  |  |  |  |  |  |
| rs1844925 | 0.09 | 0.13 | GG | 271 | 339 | **0.042** |
|  |  |  | GA | 56 | 99 |  |
|  |  |  | AA | 3 | 7 |  |
|  |  |  | Total | 330 | 445 |  |
|  |  |  |  |  |  |  |
| rs1907639 | 0.36 | 0.36 | GG | 148 | 181 | 0.731 |
|  |  |  | GA | 153 | 214 |  |
|  |  |  | AA | 52 | 52 |  |
|  |  |  | Total | 353 | 447 |  |
|  |  |  |  |  |  |  |
| rs2197205 | 0.35 | 0.34 | GG | 145 | 188 | 0.673 |
|  |  |  | GA | 138 | 205 |  |
|  |  |  | AA | 44 | 44 |  |
|  |  |  | Total | 327 | 437 |  |
|  |  |  |  |  |  |  |
| rs2399403 | 0.09 | 0.08 | TT | 303 | 389 | 0.456 |
|  |  |  | TC | 61 | 59 |  |
|  |  |  | CC | 1 | 5 |  |
|  |  |  | Total | 365 | 453 |  |
|  |  |  |  |  |  |  |
| rs4682350 | 0.01 | 0.01 | TT | 355 | 440 | 0.719 |
|  |  |  | TC | 6 | 9 |  |
|  |  |  | CC | 0 | 0 |  |
|  |  |  | Total | 361 | 449 |  |
|  |  |  |  |  |  |  |
| rs4682355 | 0.12 | 0.13 | AA | 263 | 339 | 0.647 |
|  |  |  | AG | 65 | 103 |  |
|  |  |  | GG | 8 | 6 |  |
|  |  |  | Total | 336 | 448 |  |
|  |  |  |  |  |  |  |
| rs7622025 | 0.35 | 0.36 | GG | 143 | 178 | 0.709 |
|  |  |  | GA | 175 | 218 |  |
|  |  |  | AA | 37 | 52 |  |
|  |  |  | Total | 355 | 448 |  |
|  |  |  |  |  |  |  |
| rs9811691 | 0.23 | 0.20 | GG | 213 | 285 | 0.249 |
|  |  |  | GT | 116 | 142 |  |
|  |  |  | TT | 22 | 20 |  |
|  |  |  | Total | 351 | 447 |  |
|  |  |  |  |  |  |  |
| rs9864383 | 0.35 | 0.35 | GG | 144 | 186 | 0.902 |
|  |  |  | GA | 139 | 210 |  |
|  |  |  | AA | 45 | 49 |  |
|  |  |  | Total | 328 | 445 |  |
|  |  |  |  |  |  |  |
| rs13066495 | 0.36 | 0.34 | CC | 153 | 194 | 0.416 |
|  |  |  | CT | 149 | 197 |  |
|  |  |  | TT | 55 | 54 |  |
|  |  |  | Total | 357 | 445 |  |
| *TMPRSS11E* |  |  |  |  |  |  |
| rs2603158 | 0.13 | 0.12 | TT | 249 | 347 | 0.396 |
|  |  |  | TC | 74 | 89 |  |
|  |  |  | CC | 6 | 7 |  |
|  |  |  | Total | 329 | 443 |  |
|  |  |  |  |  |  |  |
| rs2708695 | 0.36 | 0.39 | AA | 123 | 166 | 0.317 |
|  |  |  | AG | 131 | 198 |  |
|  |  |  | GG | 40 | 67 |  |
|  |  |  | Total | 294 | 431 |  |
|  |  |  |  |  |  |  |
| rs2708699 | 0.48 | 0.49 | AA | 101 | 115 | 0.820 |
|  |  |  | AG | 179 | 229 |  |
|  |  |  | GG | 87 | 103 |  |
|  |  |  | Total | 367 | 447 |  |
|  |  |  |  |  |  |  |
| rs35293564 | 0.30 | 0.28 | GG | 173 | 235 | 0.328 |
|  |  |  | GA | 153 | 182 |  |
|  |  |  | AA | 30 | 34 |  |
|  |  |  | Total | 356 | 451 |  |
| *HGF* |  |  |  |  |  |  |
| rs975360 | 0.43 | 0.41 | TT | 128 | 156 | 0.432 |
|  |  |  | TC | 153 | 217 |  |
|  |  |  | CC | 75 | 72 |  |
|  |  |  | Total | 356 | 445 |  |
|  |  |  |  |  |  |  |
| rs2040965^b^ | 0.19 | 0.19 | AA | 300 | 294 | 0.940 |
|  |  |  | AG | 147 | 140 |  |
|  |  |  | GG | 16 | 18 |  |
|  |  |  | Total | 463 | 452 |  |
|  |  |  |  |  |  |  |
| rs2158728 | 0.39 | 0.39 | CC | 123 | 165 | 0.992 |
|  |  |  | CT | 166 | 198 |  |
|  |  |  | TT | 49 | 70 |  |
|  |  |  | Total | 338 | 433 |  |
|  |  |  |  |  |  |  |
| rs2286194 | 0.17 | 0.18 | TT | 220 | 296 | 0.827 |
|  |  |  | TA | 98 | 137 |  |
|  |  |  | AA | 8 | 11 |  |
|  |  |  | Total | 326 | 444 |  |
|  |  |  |  |  |  |  |
| rs4728473 | 0.22 | 0.23 | AA | 203 | 265 | 0.542 |
|  |  |  | AT | 105 | 151 |  |
|  |  |  | TT | 18 | 26 |  |
|  |  |  | Total | 326 | 442 |  |
|  |  |  |  |  |  |  |
| rs5745687 | 0.01 | 0.02 | GG | 323 | 425 | 0.125 |
|  |  |  | GA | 8 | 20 |  |
|  |  |  | AA | 0 | 0 |  |
|  |  |  | Total | 331 | 445 |  |
|  |  |  |  |  |  |  |
| rs5745752 | 0.23 | 0.28 | GG | 209 | 233 | **0.017** |
|  |  |  | GA | 131 | 171 |  |
|  |  |  | AA | 15 | 38 |  |
|  |  |  | Total | 355 | 442 |  |
|  |  |  |  |  |  |  |
| rs6942495 | 0.38 | 0.40 | GG | 129 | 162 | 0.439 |
|  |  |  | GC | 157 | 206 |  |
|  |  |  | CC | 47 | 71 |  |
|  |  |  | Total | 333 | 439 |  |
|  |  |  |  |  |  |  |
| rs7790870 | 0.11 | 0.13 | GG | 261 | 333 | 0.200 |
|  |  |  | GC | 64 | 103 |  |
|  |  |  | CC | 5 | 8 |  |
|  |  |  | Total | 330 | 444 |  |
|  |  |  |  |  |  |  |
| rs12707453 | 0.05 | 0.07 | AA | 318 | 387 | 0.124 |
|  |  |  | AG | 34 | 53 |  |
|  |  |  | GG | 0 | 3 |  |
|  |  |  | Total | 352 | 443 |  |
|  |  |  |  |  |  |  |
| rs17155381 | 0.10 | 0.12 | TT | 286 | 348 | 0.317 |
|  |  |  | TC | 60 | 93 |  |
|  |  |  | CC | 5 | 5 |  |
|  |  |  | Total | 351 | 446 |  |
|  |  |  |  |  |  |  |
| rs35642091 | 0.14 | 0.12 | CC | 262 | 344 | 0.214 |
|  |  |  | CT | 80 | 98 |  |
|  |  |  | TT | 10 | 5 |  |
|  |  |  | Total | 352 | 447 |  |

^a^ *P* value from Armitage trend test for difference in genotype frequencies between invasive cases and controls

significant *P* values bolded

^b^ for SNPs rs2838042, rs462149, and rs2040965, 462 cases and 474 controls were available.

Abbreviations: MAF, minor allele frequency
