# Supplementary material for: Type II Transmembrane Serine Protease Gene Variants Associate with Breast Cancer
Source: PLoS One. 2014 Jul 16;9(7):e102519. doi: 10.1371/journal.pone.0102519 (PMC4100901; doi:10.1371/journal.pone.0102519)
Supplement: Table S3 — Breast cancer survival in univariate analysis (Kaplan–Meier) at the latest follow-up data point. (DOCX) [file pone.0102519.s004.docx]

**Supplemental Table S3.**

| **Gene and SNP** | **Genotype** | ***n*** | **Proportion surviving at the time (%)** | ***P* value** |
| --- | --- | --- | --- | --- |
| *uPA* |  |  |  |  |
| rs2227578 | GG | 107 | 76.0 |  |
|  | GA | 199 | 62.7 |  |
|  | AA | 100 | 62.2 | ns |
|  | GA+AA | 299 | 62.3 | 0.021 |
| *TMPRSS1* |  |  |  |  |
| rs12151195 | TT | 352 | 69.3 |  |
|  | TC | 60 | 46.8 |  |
|  | CC | 2 | 50.0 | 0.008 |
|  | TC+CC | 62 | 46.4 | 0.002 |
|  |  |  |  |  |
| rs12461158 | GG | 249 | 62.6 |  |
|  | GA | 135 | 69.0 |  |
|  | AA | 23 | 85.2 | ns |
|  | GA+AA | 158 | 72.1 | 0.05 |
| *TMPRSS2* |  |  |  |  |
| rs2070788 | TT | 163 | 59.0 |  |
|  | TC | 190 | 75.5 |  |
|  | CC | 56 | 52.6 | 0.022 |
|  |  |  |  |  |
| rs2276205 | AA | 297 | 65.0 |  |
|  | AG | 93 | 73.1 |  |
|  | GG | 11 | 33.9 | 0.038 |
|  | AA+AG | 390 | 67.0 | 0.05 |
| *TMPRSS3* |  |  |  |  |
| rs3814903 | GG | 166 | 73.7 |  |
|  | GT | 189 | 60.8 |  |
|  | TT | 50 | 62.1 | ns |
|  | GT+TT | 239 | 60.5 | 0.026 |
| *TMPRSS7* |  |  |  |  |
| rs2399403 | TT | 353 | 69.3 |  |
|  | TC | 55 | 43.0 |  |
|  | CC | 5 | 60 | 0.025 |
|  | TC+CC | 60 | 45.9 | 0.007 |
| *TMPRSS11E* |  |  |  |  |
| rs35293564 | GG | 213 | 60.2 |  |
|  | GA | 169 | 72.8 |  |
|  | AA | 29 | 59.2 | ns |
|  | GA+AA | 198 | 70.9 | 0.048 |
| *HGF* |  |  |  |  |
| rs2040965 | AA | 265 | 60.4 |  |
|  | AG | 132 | 73.7 |  |
|  | GG | 14 | 84.6 | ns |
|  | AG+GG | 146 | 74.8 | 0.035 |

Abbreviations: ns, not significant
