# Supplementary material for: Type II Transmembrane Serine Protease Gene Variants Associate with Breast Cancer
Source: PLoS One. 2014 Jul 16;9(7):e102519. doi: 10.1371/journal.pone.0102519 (PMC4100901; doi:10.1371/journal.pone.0102519)
Supplement: Table S4 — Significant associations of gene variants with clinical variables. (DOCX) [file pone.0102519.s005.docx]

**Supplemental Table S4.**

| **Gene, SNP, and clinical variable** | **Genotype 1** | **Genotype 2** |  |  |
| --- | --- | --- | --- | --- |
|  | **(ref.)** |  |  |  |
|  | ***n*** | ***n*** | ***P* value**^a^ | **OR (95% CI)**^b^ |
| ***uPA*** |  |  |  |  |
| **rs2227578** | **GG** | **GA+AA** | 0.039^a^ |  |
| Nodal status |  |  |  |  |
| Negative | 74 | 168 |  | ref. |
| Positive | 30 | 126 | 0.013 | 1.850 (1.141–2.999) |
| ***TMPRSS1*** |  |  |  |  |
| **rs2305747** | **TT** | **TC+CC** | 0.038^a^ |  |
| ER status |  |  |  |  |
| Negative | 61 | 28 |  | ref. |
| Positive | 170 | 134 | 0.035 | 1.717 (1.040–2.835) |
|  |  |  |  |  |
| **rs8107142** | **GG** | **GA+AA** | 0.021^a^ |  |
| PR status |  |  |  |  |
| Negative | 109 | 39 | 0.017 | 1.834 (1.114–3.019) |
| Positive | 205 | 40 |  | ref. |
|  |  |  |  |  |
| **rs12151195** | **TT** | **TC+CC** | 0.017^a^ |  |
| PR status |  |  |  |  |
| Negative | 118 | 31 | 0.015 | 1.993 (1.146–3.467) |
| Positive | 220 | 29 |  | ref. |
|  |  |  |  |  |
| **rs12461158** | **GG** | **GA+AA** |  |  |
| HER2 status |  |  |  |  |
| Positive | 36 | 12 |  | ref. |
| Negative | 195 | 137 | 0.034 | 2.108 (1.058–4.197) |
| ***TMPRSS2*** |  |  |  |  |
| **rs462471** | **GG** | **GA+AA** |  |  |
| PR status |  |  |  |  |
| Negative | 62 | 84 | 0.047 | 1.518 (1.005–2.294) |
| Positive | 130 | 116 |  | ref. |
|  |  |  |  |  |
| **rs734056** | **CC+CA** | **AA** | 0.025^a^ |  |
| PR status |  |  |  |  |
| Negative | 132 | 14 |  | ref. |
| Positive | 200 | 46 | 0.017 | 2.169 (1.147–4.102) |
|  |  |  |  |  |
| **rs2276205** | **AA** | **AG+GG** | 0.016^a^ |  |
| PR status |  |  |  |  |
| Negative | 116 | 29 |  | ref. |
| Positive | 169 | 71 | 0.039 | 1.680 (1.027–2.750) |
|  |  |  |  |  |
| **rs3787950** | **TT** | **TC+CC** |  |  |
| PR status |  |  | 0.003^a^ |  |
| Negative | 136 | 12 | 0.005 | 5.272 (1.668–16.667) |
| Positive | 239 | 4 |  | ref. |
| ER status |  |  | 0.014^a^ |  |
| Negative | 82 | 8 | 0.013 | 3.598 (1.310–9.878) |
| Positive | 295 | 8 |  | ref. |
|  |  |  |  |  |
| **rs8127664** | **CC+CT** | **TT** | 0.019^a^ |  |
| Tumor stage |  |  |  |  |
| I | 155 | 5 |  | ref. |
| II, III | 210 | 23 | 0.015 | 3.395 (1.263–9.129) |
|  |  |  |  |  |
| **rs9984523** | **CC** | **CT+TT** | 0.004^a^ |  |
| Tumor size |  |  |  |  |
| T1 | 184 | 33 |  | ref. |
| T2, T3, T4 | 139 | 54 | 0.0018 | 2.166 (1.333–3.521) |
| ***TMPRSS3*** |  |  |  |  |
| **rs186531** | **CC** | **CT+TT** |  |  |
| Tumor grade |  |  |  |  |
| Low | 85 | 26 | 0.045 | 2.076 (1.018–4.233) |
| Medium | 143 | 48 | 0.013 | 2.278 (1.190–4.360) |
| High | 95 | 14 |  | ref. |
|  |  |  |  |  |
| **rs225310** | **GG** | **GT+TT** |  |  |
| Tumor stage |  |  | 0.003^a^ |  |
| I | 49 | 112 | 0.001 | 1.995 (1.308–3.043) |
| II, III | 110 | 126 |  | ref. |
| Tumor size |  |  | 0.008^a^ |  |
| T1 | 73 | 143 | 0.004 | 1.804 (1.211–2.687) |
| T2, T3, T4 | 93 | 101 |  | ref. |
|  |  |  |  |  |
| **rs1078272** | **TT** | **TA+AA** |  |  |
| Histological type |  |  | 0.019^a^ |  |
| (Medullary and) other | 35 | 38 |  | ref. |
| Ductal | 124 | 140 | 0.883 | Ns |
| Lobular | 19 | 50 | 0.013 | 2.424 (1.204–4.880) |
| Tumor stage |  |  | 0.040^a^ |  |
| I | 58 | 102 | 0.021 | 1.628 (1.078–2.458) |
| II, III | 112 | 121 |  | ref. |
|  |  |  |  |  |
| **rs2839489** | **TT** | **TG+GG** |  |  |
| HER2 status |  |  |  |  |
| Positive | 32 | 14 |  | ref. |
| Negative | 174 | 158 | 0.031 | 2.076 (1.069–4.032) |
| Tumor stage |  |  | 0.005^a^ |  |
| I | 72 | 88 | 0.001 | 1.964 (1.305–2.955) |
| II, III | 143 | 89 |  | ref. |
|  |  |  |  |  |
| **rs3814899** | **GG** | **GC+CC** |  |  |
| Histological type |  |  |  |  |
| (Medullary and) other | 42 | 33 |  | ref. |
| Ductal | 176 | 88 | 0.090 | NS. |
| Lobular | 52 | 16 | 0.011 | 0.392 (0.190–0.860) |
|  |  |  |  |  |
| **rs13047838** | **CC** | **CT+TT** |  |  |
| Histological type |  |  | 0.029^a^ |  |
| (Medullary and) other | 42 | 34 |  | ref. |
| Ductal | 188 | 74 | 0.007 | 0.486 (0.287–0.823) |
| Lobular | 41 | 28 | 0.613 | ns |
| Tumor stage |  |  | 0.009^a^ |  |
| I | 94 | 69 | 0.003 | 1.915 (1.254–2.926) |
| II, III | 167 | 64 |  | ref. |
| ***TMPRSS7*** |  |  |  |  |
| **rs7622025** | **GG** | **GA+AA** |  |  |
| Tumor grade |  |  |  |  |
| Low | 30 | 80 | 0.030 | 1.875 (1.064–3.305) |
| Medium | 81 | 106 | 0.733 | Ns |
| High | 45 | 64 |  | ref. |
| ***TMPRSS11E*** |  |  |  |  |
| **rs2708699** | **AA+AG** | **GG** |  |  |
| ER status |  |  |  |  |
| Negative | 61 | 29 | 0.034 | 1.755 (1.043–2.953) |
| Positive | 240 | 65 |  | ref. |
| ***HGF*** |  |  |  |  |
| **rs2286194** | **TT** | **TA+AA** |  |  |
| Tumor grade |  |  |  |  |
| Low | 83 | 25 |  | ref. |
| Medium | 114 | 72 | 0.007 | 2.097 (1.227–3.583) |
| High | 68 | 41 | 0.022 | 2.002 (1.108–3.617) |
|  |  |  |  |  |
| **rs6942495** | **GG+GC** | **CC** |  |  |
| Nodal status |  |  | 0.002^a^ |  |
| Negative | 211 | 28 |  | ref. |
| Positive | 117 | 36 | 0.002 | 2.319 (1.347–3.991) |
| Tumor size |  |  | 0.00009^a^ |  |
| T1 | 189 | 22 |  | ref. |
| T2, T3, T4 | 145 | 44 | 0.0007 | 2.607 (1.496–4.544) |
| Tumor stage |  |  | 0.0004^a^ |  |
| I | 142 | 16 |  | ref. |
| II, III | 181 | 48 | 0.006 | 2.345 (1.283–4.318) |
|  |  |  |  |  |
| **rs35642091** | **CC** | **CT+TT** |  |  |
| ER status |  |  | 0.007^a^ |  |
| Negative | 55 | 32 | 0.002 | 2.244 (1.339–3.761) |
| Positive | 243 | 63 |  | ref. |
| PR status |  |  |  |  |
| Negative | 102 | 45 | 0.025 | 1.712 (1.071–2.735) |
| Positive | 194 | 50 |  | ref. |

^a^ *P* value for overall comparison from χ^2^ test for differences in frequencies when genotype is in three categories; other *P* values from logistic regression

^b^ OR (odds ratio) and 95% CI (confidence interval) values for association

Abbreviations: ref. , reference category in the logistic regression analysis; ns, not significant
